# Processing methodology of global anthropogenic emissions for air quality modeling

**DOI:** 10.1016/j.mex.2021.101505

**Published:** 2021-08-31

**Authors:** Ernesto Pino-Cortés

**Affiliations:** Escuela de Ingeniería Química, Pontificia Universidad Católica de Valparaíso, Ave Brasil 2162, Valparaíso, Chile

**Keywords:** NCO, CAMS, SMOKE, Simulation, Emission inventory

## Abstract

The Global Emissions Initiative (GEIA) stores and offers global datasets of emission inventories developed in the last 30 years. One of the most recently updated global datasets covering anthropogenic source emissions is the Copernicus Atmosphere Monitoring Service (CAMS). This study applied NetCDF Command Operator (NCO) software to preprocess the anthropogenic sources included in the CAMS datasets and converted those files as an input in the Sparse Matrix Operator Kerner Emissions (SMOKE) model for future air quality modeling. As a result, six steps were applied to obtain the required file format. The case of the central coast in Chile was analyzed to compare the global database and official reports for the on-road transport sector. As a result, some differences were shown in the most populated locations of the domain of analysis. The rest of the zones registered similar values. The methodology exposed in this report could be applied in any other region of the planet for air quality modeling studies. The development of global datasets such as CAMS is useful for hemispheric analysis and could bring an estimation on the mesoscale. It represents an opportunity for those locations without official reports of non-updated data.

• This study applied NCO commands available for the preprocessing of the CAMS dataset files.
• The emissions and temporal profile registered in CAMS datasets must be compared to official reports of transport sectors.
• The development of global datasets such as CAMS is useful for hemispheric analysis and could bring an estimation on the mesoscale.

## Graphical abstract

**Figure:**
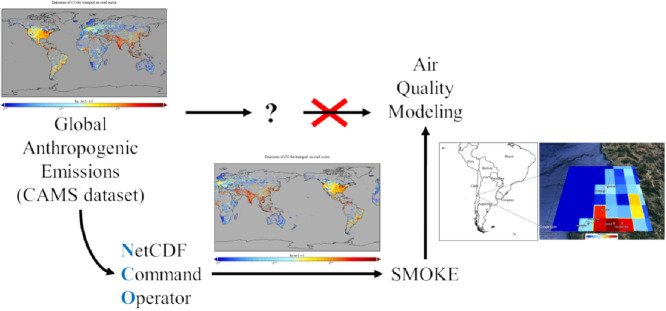

Specifications table

**Table:** 

Subject Area:	Environmental Science
More specific subject area:	*Air emission inventory of anthropogenic sources*
Method name:	*Preprocessing steps of CAMS-GLOB-ANT datasets to input into SMOKE model.*
Name and reference of original method:	*C. S. Zender, Analysis of self-describing gridded geoscience data with netCDF Operators (NCO). Environmental Modelling & Software, 23(10–11) (2008) 1338–1342, doi:.org/10.1016/J.ENVSOFT.2008.03.004.*
Resource availability:	*The files of CAMS datasets are hosted on the GEIA website (*http://www.geiacenter.org*). The SMOKE codes can be downloaded at*www.cmascenter.org

## Background

The most accurate and absolute air emission inventory estimation is crucial to achieving an air quality simulation [1]. Some countries have established datasets with this information at a high level of detail, improving and enhancing studies for better environmental policy at local, regional and national levels. Unfortunately, there are many regions with unclear or undefined emission inventories, avoiding air quality models in those locations.

In the last 30 years, various global datasets of emission inventories have been developed for different sources and covering specific periods of analysis. Today, the Global Emissions Initiative (GEIA) stores and offers those datasets. It represents an opportunity for those locations without official reports of non-updated data. More information about the organization's mission and goals can be found on its website (http://www.geiacenter.org). One of the most recently updated global datasets covering anthropogenic source emissions is the Copernicus Atmosphere Monitoring Service (CAMS) developed by the European Centre for Medium-Range Weather Forecasts (ECMWF) on behalf of the European Commission.

CAMS datasets are a compiled emission inventory developed for the years 2000–2020 for many atmospheric compounds. The anthropogenic sources include 12 sectors, and the spatial resolution is 0.1°. The methodology of the emission inventory estimation can be accessed in a published report [2]. The files of CAMS datasets are hosted on the GEIA website. Additionally, they are processed with the Network Common Data Form (NetCDF) format and are available upon user request. The instructions for downloading the desired files are sent by email to the registered user.

To date, there have been no published reports on the application of CAMS datasets as an input to air quality models. One of the main causes could be the lack of information about its processing. The purpose of this study is to expose a methodology for CAMS dataset processing and convert those files as an input in the Sparse Matrix Operator Kerner Emissions (SMOKE) model [3]. SMOKE is the preprocessor of the emission inventory for air quality models such as CMAQ [4] and CAMx [5]. The steps reported could be applied in future air quality modeling studies on regions where the emission inventory is not defined or not developed at all.

## Method details

The CAMS-GLOB-ANT datasets contain monthly emissions for anthropogenic sources. In this study, the on-road transport (tro) sector was analyzed. All files were processed using NetCDF Command Operator (NCO) software. NCO is designed for the analysis and modification of gridded data stored in NetCDF format [6]. This software was released in 2008, and many research studies have been developed by using this tool since then. NCO has different types of commands for reading, writing, interpolating and averaging [7].

In this study, the first step was the extraction of the emissions for one month, as shown in Table 1. If the user downloads more than one month in a unique file, the first month's counter must start at zero. For example, in this study, the total emissions for 2018 were downloaded, so the counter for August emissions was the number seven. The counter for the rest of the monthly emissions must be according to the item in an ordered list. While the user downloads one file with one month of data, this step is not needed.

**Table 1:** Main steps for processing the CAMS dataset to obtain the required format as an input in SMOKE.

Step	command	Example
1	ncks	ncks -O -d time," number related to the month of analysis" CAMS_file fileSTEP1_out
2	ncwa	ncwa -O -a time fileSTEP1_out fileSTEP2_out
3	ncks	ncks -C -O -x -v time fileSTEP2_out fileSTEP3_out
4	ncks	ncks -O -3 fileSTEP3_out fileSTEP4_out
5	ncks	ncks -O -v tro –msa -d lon,0.,180. -d lon,-180.,0. fileSTEP4_out fileSTEP5_out
6	ncks	ncap2 -O -s 'where(lon < 0) lon=lon+360′ fileSTEP6_out file_for_SMOKE.nc

After the extraction of the desired monthly data, the file format must be modified. The second step deletes the attribute named "time" in the extracted file in step 1. Next, the variable "time" is also deleted in step 3. SMOKE does not read gridded emission inventory data with that attribute and variable [3], which is why they must be extracted in steps 2 and 3. The next step (4) changes the file format to NetCDF type 3, which is a SMOKE requirement for these gridded emission data. Steps 2-4 are also mentioned in the methodology reported by Pino-Cortés et al. [8].

**Fig 1:**
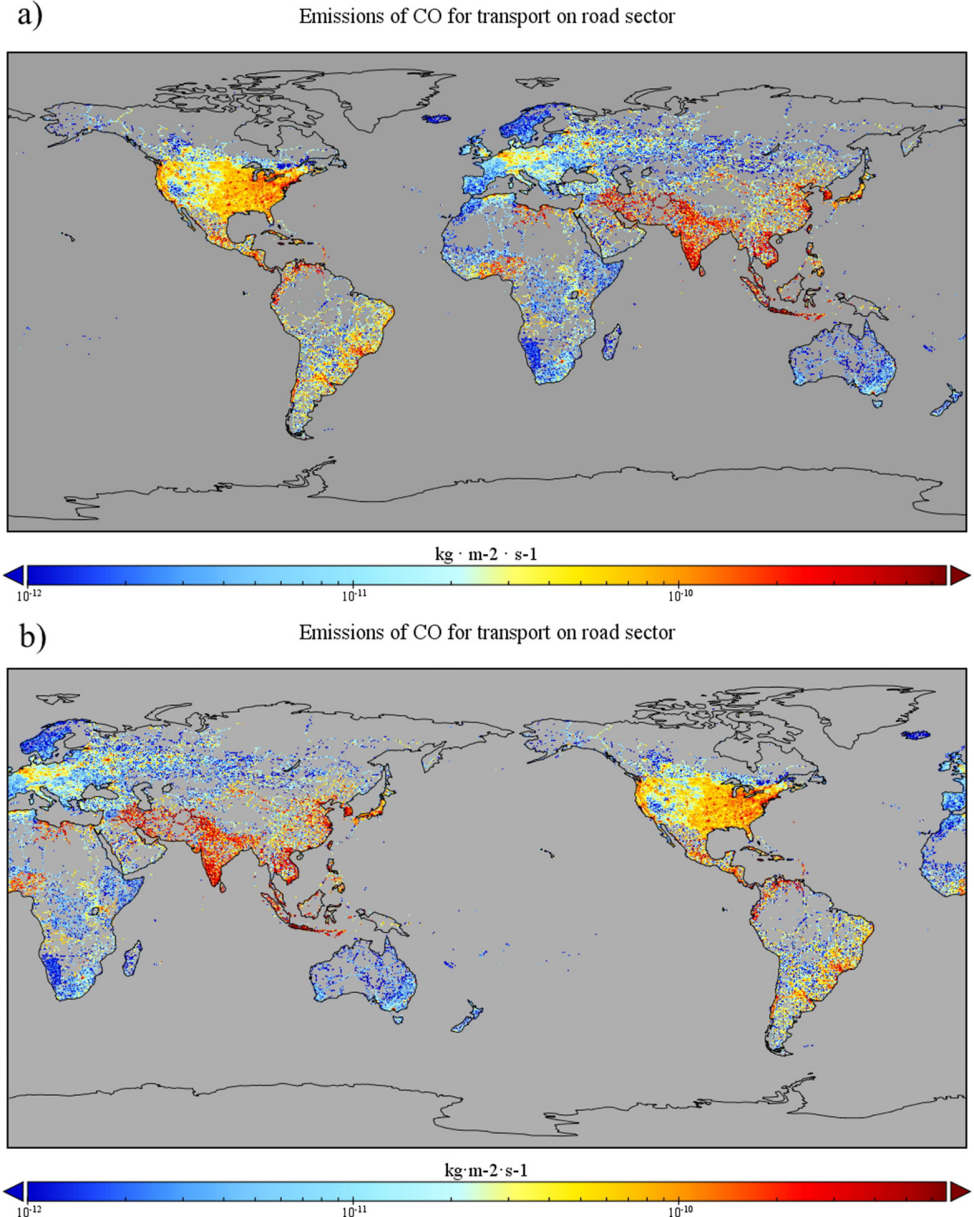
CO emissions of CAMS datasets. a) original format. b) processed using NCO in this study.

Finally, steps 5 and 6 modify the attribute "longitude". The original file of the CAMS-GLOB-ANT datasets has longitudes ordered from 180° to -180°, as shown in Fig. 1 (top). However, this attribute must be relocated from 0° to 360°, with 0° longitude relative to the original column. Fig. 1 (downside) shows the modification of step 6. The output file from step 6 has all the requirements to input into the SMOKE model. All steps presented in Table 1 could be replicated for all sectors included in the CAMS-GLOB-ANT datasets.

## Method validation

The Puchuncavi-Quintero-Concon industrial complex is located on the central coast of Chile in communes with the same names. Currently, this area is saturated by particulate matter, although there is also monitoring of sulfur dioxide (SO_2_), ozone (O_3_), nitrogen dioxide (NO_2_) and carbon monoxide (CO) at different air quality monitoring stations. In this industrial zone, the inventory of emissions from industrial sources is available and detailed as an individual point source at the Chilean Pollutant Release and Transfer Registers (PRTR) website (https://retc.mma.gob.cl/). Otherwise, annual transport emissions are reported at the commune level, but they are limited to specific areas, and gridded georeferenced data are not available. This is the main limitation for the evaluation of this source emission in studies of air quality.

This study applied the CAMS-GLOB-ANT datasets for the identification and analysis of emissions from the transport sector. The Weather Research and Forecast (WRF) model version 4.1.2 was used to create a domain of analysis with 73 × 73 grid cells and 1 km of horizontal resolution. The WRF model was run for 1 h, and the output file was processed in the computational module Meteorology-Chemistry Interface Processor (MCIP) [9]. MCIP creates four files with the georeferenced information required by the SMOKE model and reduces 3 grid cells for each side of the domain, generating a new domain with 70 × 70 grid cells in this study.

The monthly processed files as described in the previous section were input into SMOKE as gridded data.

**Fig. 2:**
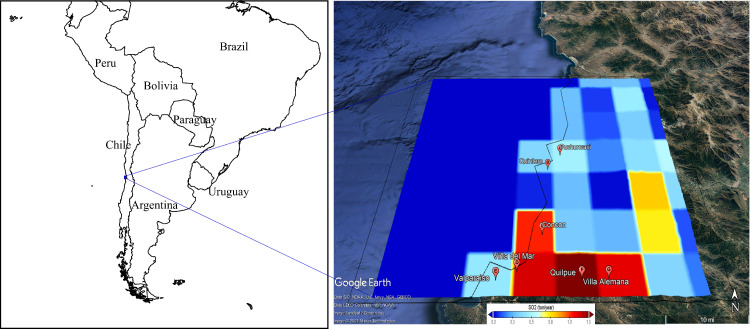
Representation of the SO_2_ emissions from the SMOKE output file.

The spatial distribution of the emissions processed in SMOKE is shown in Fig. 2. The letters represent the communes' location in the domain of analysis. The gridded cells positioned in the ocean are explained by the resolution of the CAMS dataset files. However, the emissions processed in SMOKE can be considered acceptable and a preliminary estimation for future air quality modeling.

One of the main uncertainties for emission inventory simulation is the temporal profile of the emissions. The SMOKE output files bring the monthly fraction of transport emissions, as shown in Fig. 2. In this case, three different registries were obtained for different communes in the zone of analysis.

**Fig. 3:**
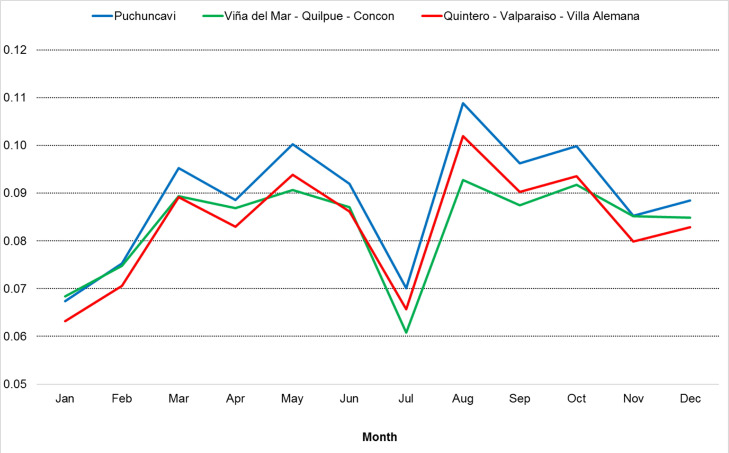
Monthly temporal fraction of the emissions in 2018.

The highest emissions occurred during August 2018 for all communes. In contrast, the lowest fractions were observed in January, February and July, when the holiday season is present in Chile. The temporal profile obtained using CAMS datasets is reasonable except for Viña del Mar and Valparaiso. These communes received many tourists during holidays every year, impacting the transport sector and increasing traffic on urban streets. In January and February, the monthly profile must be higher than the rest of the year. The information shown in Fig. 3 could help future studies of emission inventories about this anthropogenic source in Chile.

The CAMS dataset registries were compared to the official report from PRTR in Chile [10]. As shown in Table 2, most of the emissions recorded in Valparaiso and Viña del Mar from the global database are lower than the government estimation in Chile. Both communes are the most populated in the region and receive many flotant people every day. Otherwise, the records for Quilpue, Villa Alemana and Concon are comparable, except for CO in Quilpue, where the CAMS dataset registered more than twice the official report. These zones are considered housing regions, and their emissions from the transport sector are lower than those reported in Valparaiso and Viña del Mar. Finally, the emissions recorded in Quintero from the global database are 3 times higher than those in the national registries. This could be explained because the industrial sector is the most current activity in this commune. Finally, there are no official emission records for the Puchuncavi commune, and the values from the CAMS dataset could be helpful for air quality modeling in this zone. It is remarkable to distinguish that the emissions from the official report are estimated using mobile models.

**Table 2:** Comparison of the annual emissions (ton/year) from CAMS datasets and official reports in Chile.

Commune	Sulfur dioxide		Carbon Monoxide		Methane	
	CAMS	Official report	CAMS	Official report	CAMS	Official report
Valparaiso	0.439	3.392	2260	4057	4.610	24.800
Viña del Mar	1.148	4.997	5713	5895	11.757	36.475
Quilpue	1.263	1.530	4466	2000	8.925	10.918
Villa Alemana	1.110	1.173	3674	1256	7.470	7.867
Concon	1.036	0.627	2154	1353	4.437	5.968
Quintero	0.355	0.142	961	217	1.876	0.622
Puchuncavi	0.489	-	1522	-	3.170	-

Supplementary material and/or Additional information: A bash script file is included and named as conversion_transport.sh. This file resumes the steps exposed here and could be applied in a linux operating system with some examples emission inventories files included.

## Declaration of Competing Interest

The author declare that he has no known competing financial interests or personal relationships that could have appeared to influence the work reported in this paper.

## Appendix Supplementary materials

Image, application 1
